# The complete mitochondrial genome of sponge *Pseudosuberites* sp. (Demospongiae, Suberitida, Suberitidae) from Dokdo, Republic of Korea (East Sea)

**DOI:** 10.1080/23802359.2019.1692731

**Published:** 2019-11-21

**Authors:** Cheol Yu, Dong Won Kang, Hana Kim, Hyung June Kim

**Affiliations:** a Department of Taxonomy and Systematics, National Marine Biodiversity Institute of Korea, Seocheon-gun, Chungcheongnam-do, Republic of Korea;; b Department of Oceanography and Ocean Environmental Sciences, Chungnam National University, Yuseong-gu, Daejeon, Republic of KoreaHKHyung June Kim;; c Department of Biological Sciences, Inha University, Nam-gu, Incheon, Republic of Korea

**Keywords:** Mitochondrial genome, *Pseudosuberites*, Suberitidae, demosponge

## Abstract

The mitogenome of *Pseudosuberites* sp. (Suberitida, Suberitidae) has been determined first in the genus *Pseudosuberites*. Assembled mitogenome was 23,502 bp in length, including 14 protein-coding genes, 25 transfer RNA, and 2 ribosomal RNA genes. The order and structure are the same as those of other species belonging to the same family Suberitidae. *Pseudosuberites* sp. was clustered with *Suberites domucula* within the family Suberitidae. The mitogenome of *Pseudosuberites* sp. will be valuable for inferring phylogenetic relationships among members of suberitids.

*Pseudosuberites* sp. is encrusting species and inhabits ∼20 m depth in the subtidal zone. The genus *Pseudosuberites* includes 18 species globally (van Soest et al. 2019). The members of this genus are characterized with tangential ecotosomal skeleton of tylostyles over a confused choanosomal skeleton of tylostyles bundles and individual spicules (Hooper and van Soest 2002). The species analyzed in this study was identified as a new species belonging to the genus *Pseudosuberites*. Until now, the only one species (*Suberites domuncula*, AM690374) of suberitids has been reported, for complete mitochondrial genomes (mitogenome) (Lukić-Bilela et al. 2008). We report the complete mitogenome of *P.* sp. for the first time in the genus *Pseudosuberites*, and it will be valuable information for further study on molecular taxonomy and phylogeny of this taxon.

Specimens of *Pseudosuberites* sp. were collected from the subtidal zone of Dokdo, Republic of Korea via SCUBA diving in the East Sea (1 June 2019, 37°14′35.13″N 131°51′43.83″E). Voucher specimens were deposited in the National Marine Biodiversity Institute of Korea (MABIK IV00166796). Genomic DNA was extracted from the tissue, and mitogenome sequences were analyzed by application of Illumina Hiseq2000 sequencing platform (Macrogen, Seoul, Korea). Sequences were assembled and annotated, in comparison with the previously reported mitogenome sequences of a suberitid species using Geneious 9.1.8 (www.geneious.com). Additionally, we used the mitochondrial genome annotation server (Bernt et al. 2013), and tRNAscan-SE server (Lowe and Chan 2016) for annotation. The maximum-likelihood (ML) tree was constructed, to investigate molecular taxonomic position of these species using GTR + G model in MEGA version X (Nei and Kumar 2000; Kumar et al. 2018), and dataset were used with nucleotide sequences of 14 protein-coding genes (PCGs) from mitogenomes of other eight heteroscleromorphan sponge species.

The complete mitogenome of *Pseudosuberites* sp. (GeneBank accession number MN547324) is 23,502 bp in length, containing 14 PCGs, 2 ribosomal RNAs, and 25 transfer RNAs (tRNAs). Overall nucleotide base composition of *Pseudosuberites* sp. is 30.6% A, 13.8% C, 20.5% G, 35.1% T, respectively, revealing high A + T bias (65.7%) similar to the other suberitid sponges. All PCGs use typical ATG as start codon. Ten PCGs (*cox1, nad1, nad5, cox2, atp6, cox3, atp9, nad4, nad6,* and *nad3*) use TAA as the stop codon, while four (*nad2, atp8, cob,* and *nad4l*) gene have TAG. The length of tRNA genes range from 70 to 83 bp, and 24 tRNAs have the typical clover leaf structure except only one tRNA^Ser^_UCN_ with v-loop.

In ML tree, the *Pseudosuberites* sp. was clustered with *Suberites domucula* belongs in the same family Suberitidae (Figure 1). Subertids are closely related with halichondriids, as well as support for previously phylogenetic studies inferred using nuclear and mitochondrial genes (Morrow et al. 2012). Morphologically, subertids and halichondriids share the presence of a confused choanosomal skeleton (Erpenbeck et al. 2012).

**Figure 1.**
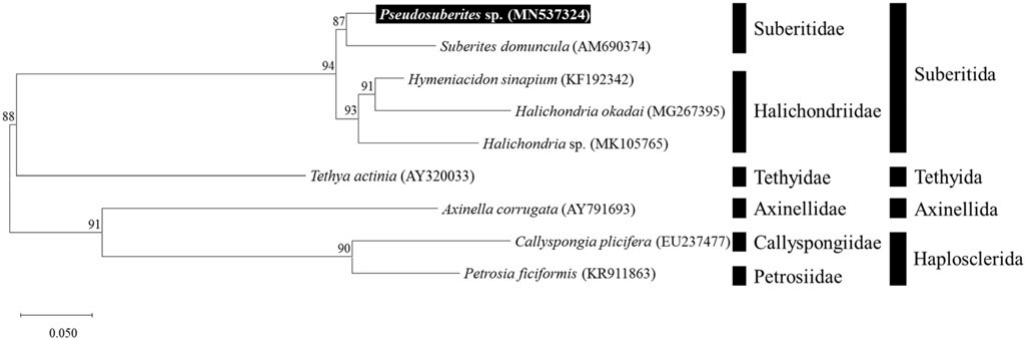
ML tree based on the PCGs of *Pseudosuberites* sp. with family Suberitidae and other species. Numbers above the branches indicate ML bootstrap values from 1000 replications.
